# Tibial Spine Avulsion Fractures in Paediatric Patients: A Systematic Review and Meta-Analysis of Surgical Management

**DOI:** 10.3390/children11030345

**Published:** 2024-03-14

**Authors:** Mehak Chandanani, Raian Jaibaji, Monketh Jaibaji, Andrea Volpin

**Affiliations:** 1 School of Medicine, Medical Sciences and Nutrition, University of Aberdeen, Aberdeen AB25 2ZD, UK; m.chandanani.21@abdn.ac.uk; 2 University College London, London WC1E 6BT, UK; raian.jaibaji.19@ucl.ac.uk; 3 Health Education North East, Newcastle upon Tyne NE15 8NY, UK; monketh.jaibaji@nhs.net; 4 NHS Grampian, Aberdeen AB15 6RE, UK

**Keywords:** tibial spine avulsion, paediatric fracture, arthroscopy

## Abstract

Background: Tibial spine avulsion fractures (TSAFs) account for approximately 14% of anterior cruciate ligament injuries. This study aims to systematically review the current evidence for the operative management of paediatric TSAFs. Methods: A search was carried out across four databases: MEDLINE, Embase, Scopus, and Google Scholar. Studies discussing the outcomes of the surgical management of paediatric TSAFs since 2000 were included. Results: Of 38 studies included for review, 13 studies reported outcomes of TSAF patients undergoing screw fixation only, and 12 studies used suture fixation only. In total, 976 patients underwent arthroscopic reduction and internal fixation (ARIF), and 203 patients underwent open reduction and internal fixation (ORIF). The risk of arthrofibrosis with the use of ARIF (*p* = 0.45) and screws (*p* = 0.74) for TSAF repair was not significant. There was a significantly increased risk of knee instability (*p* < 0.0001), reoperation (*p* = 0.01), and post-operative pain (*p* = 0.007) with screw fixation compared to sutures. Conclusions: While the overall benefits of sutures over screws and ARIF over ORIF are unclear, there is clear preference for ARIF and suture fixation for TSAF repair in practice. We recommend large-scale comparative studies to delineate long-term outcomes for various TSAF fixation techniques.

## 1. Introduction

Tibial eminence fractures (TEFs), also referred to as tibial spine avulsion fractures (TSAFs) and anterior cruciate ligament (ACL) avulsion fractures, have been defined as bony avulsions of the ACL from its point of insertion on the intercondylar eminence of the tibia [1]. These injuries are most common in skeletally immature paediatric patients, accounting for approximately 14% of ACL injuries across paediatric and adult populations overall [2].

TSAFs are commonly sports-related injuries, with higher occurrence in sports such as cycling and skiing. The higher occurrence rates in children have been attributed to many causes, including the greater degree of elasticity in ligaments of young people and the weakness of incomplete ossification of the tibial eminence in relation to ACL fibres in this population [3].

TSAFs are classified in accordance with the Meyers and McKeevers (MM) classification system into type I, type II, and type III [4]. This was later modified by Zaricznyj, with the addition of type IV [5]. Details of this modified MM classification can be found in Table 1. Other classification systems include the Green Tuca classification, which uses a quantitative, magnetic resonance imaging (MRI)-based system to guide the treatment and management of TSAFs, as compared to plain radiograph evaluation in the MM system [6]. However, both systems have shown good inter-reliability [6].

There is broad consensus about the non-operative management of MM type I TSAFs, using casting and immobilization for 6–12 weeks, followed by a gradual transition to weight bearing and range of motion exercises [7]. The use of operative management to treat type II fractures is controversial, with a lack of consensus. Operative management is considered for types II, III, and IV TSAFs with unsuccessful closed reduction [7].

Multiple techniques exist for the operative fixation, which include arthroscopic (ARIF) and open (ORIF) approaches. There is a lack of consensus in the literature regarding the best method of fixation. Fixation materials most commonly include sutures, K-wires, and screws. With varying degrees of complications—including arthrofibrosis, non-union, mal-union, instability, and pain—with different procedures, there is currently a lack of consensus around the indications for use of different materials and approaches [8].

This study aims to systematically review the evidence base regarding the operative management of TSAFs in a paediatric population, with a focus on various approaches, subjective and objective outcomes, and complication rates. All the reporting is in accordance with the Preferred Reporting Items for Systematic Reviews and Meta-Analysis (PRISMA) guidelines.

## 2. Materials and Methods

### 2.1. Eligibility Criteria

The following inclusion criteria were applied: (i) Studies conducted after the year 2000 (ii) assessing outcomes of surgical management (including ORIF and ARIF approaches) of TSAFs (iii) in a skeletally immature population. Literature reviews, technical notes, cadaveric studies, conference abstracts, and case reports were excluded. Studies were only included if they had a minimum of five patients.

### 2.2. Information Sources and Search Strategy

A literature search was carried out on 9 January 2024 across four databases, namely MEDLINE (Ovid), Embase, Scopus, and Google Scholar. The search was carried out using relevant medical subject headings (MeSH) and synonyms for the following keywords: (‘Tibial’ AND ‘Spine’ AND ‘Fracture’) AND ‘Surgical’ AND ‘Paediatrics’. Further details of the search strategy can be found in Appendix A. Articles with no fully published English Language text were excluded; however, a language restriction was not applied to the search itself. Fully published articles for conference abstracts were sought and included. Reference lists of systematic and literature reviews were also searched for relevant texts for inclusion. The search results were transferred to the Rayyan systematic review software for de-duplication and screening [9].

### 2.3. Selection Process

Following removal of duplicates, all search results were screened by two independent reviewers in two stages: (i) title and abstract stage, (ii) full manuscript review according to pre-defined inclusion and exclusion criteria. Reviewers were blinded to each other’s decisions during the screening process. Decisions were adjudicated at the end of each stage, and any discrepancies were resolved through discussion and consensus in the presence of a third reviewer.

### 2.4. Data Collection Process and Data Items

To ensure standardization of the data collection process, a data extraction form was designed. Data was extracted under the following domains: (i) Study characteristics—study design, author conflicts of interest, year of publication, country of origin, and level of evidence; (ii) Participant characteristics—number of participants, mean age, MM classification of fracture, surgical technique used, materials used, mean follow-up time; and (iii) Outcomes—pre- and post-surgery outcome scores (including the International Knee Documentation Committee (IKDC) scores and Lysholm scores). Data was independently extracted from the included texts simultaneously by two reviewers. Upon completion, agreement between reviewers was checked through discussion in the presence of an adjudicator and consensus was reached following any discrepancies.

### 2.5. Study Risk of Bias Assessment

The quality of studies was assessed using the Methodological Index for Non-Randomized Studies (MINORS) criteria for non-randomized studies [10]. All quality assessment was conducted by two independent reviewers. The reviewers were blinded to each other’s decisions until completion. Upon completion, concordance was checked between reviewers, and any discrepancies were resolved by discussion in the presence of a third adjudicator.

### 2.6. Data Synthesis and Measures of Effect

Data was presented in the form of four tables, namely: (i) Study Characteristics, (ii) Critical Appraisal, (iii) Population Characteristics, and (iv) Outcomes. Analysis of data was presented narratively. Statistical analysis was conducted using a random-effects model, with the use of Odds Ratios (OR), 95% Confidence Intervals (95% CI), and *p* values. A random effects model was used to control for unobserved heterogeneity. A *p* value of <0.05 was determined to be statistically significant. All statistical analysis was done using RevMan v 5.4.1.

### 2.7. Heterogeneity and Subgroup Analysis

Heterogeneity was measured using the I^2^ statistic, where an I^2^ of 0%, 25%, 50%, and 75% correspond to no, low, moderate, and high levels of heterogeneity, respectively.

## 3. Results

### 3.1. Search Results and Study Characteristics

**Figure 1:**
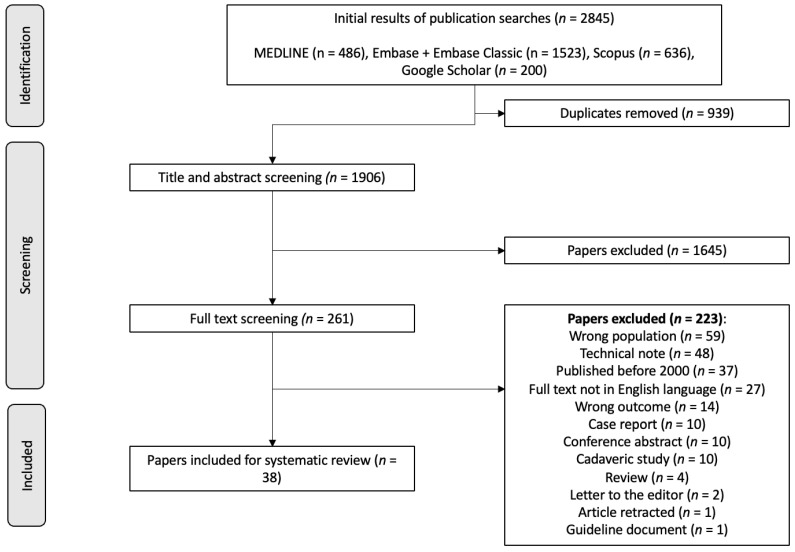
PRISMA Flow Diagram for Systematic Reviews. Summary of search screening progress.

The process of selection and inclusion of studies has been detailed in Figure 1. Of 2845 studies initially retrieved from the database search, 1906 studies were included for title and abstract screening after de-duplication. A total of 261 studies were screened by full text for inclusion within the study, of which 38 studies were found eligible for inclusion. The characteristics of the included studies have been detailed in Table 2.

### 3.2. Critical Appraisal

The quality of evidence was generally low. The main reasons for this include the retrospective nature of studies, the lack of control groups, and short follow-up periods. Studies also failed to calculate prospective sample sizes. The MINORS critical appraisal has been reported in Table 3.

### 3.3. Population Characteristics

Across 38 studies, a total of 1237 participants were included for TSAF repair. Of these, 34 patients had MM type I TSAFs (2.7%), 473 had MM type II TSAFs (38.2%), 637 had MM type III TSAFs (51.4%), and 37 had MM type IV TSAFs (2.9%). Three studies did not report the classification of their participants’ TSAFs, accounting for 59 uncategorized participants (4.7%) [19,37,41]. A total of 976 TSAF patients were treated using ARIF (78.9%), 203 patients were managed using ORIF (16.4%), 54 patients were managed conservatively using closed reduction and casting (4.3%), and 4 patients were managed using a mixed approach (0.3%). A detailed description of participant characteristics of individual studies can be found in Table 4.

### 3.4. Screw vs. Suture Fixation

Treatment with screws was reported for 333 cases, while 313 cases used sutures. A total of 21 cases used both screws and sutures. Thirteen studies reported outcomes with the use of screws only, of which ten studies used ARIF [18,21,24,25,26,29,30,36,39,42], one study used ORIF [33], and two studies used both ARIF and ORIF [43,47]. Twelve studies reported outcomes with the use of sutures only, of which nine studies used ARIF [11,13,16,31,32,37,45,46,48], and three studies used both ARIF and ORIF [17,19,38]. Four studies directly compared the use of sutures with the use of screws [12,14,22,34].

Of patients undergoing ARIF, 5 patients had complications with suture fixation (5/172, 2.9%), and 21 patients had complications with screw fixation (21/161, 13.0%); the difference was statistically significant (OR 5.01 [95% CI 2.0–12.4], p.0006). The study outcomes have been detailed in Table 5, and the related complications have been detailed in Table 6.

### 3.5. Screw vs. Suture Risk of Arthrofibrosis

**Figure 2:**
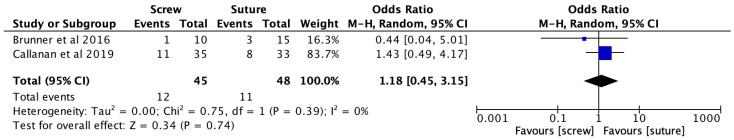
Forest Plot Comparison of Screw vs. Suture for Arthrofibrosis [12,14].

After pooling the outcomes of the studies comparing screw and suture interventions [12,14], the results revealed an increased risk of screw fixation over suture fixation for development of arthrofibrosis however, this did not reach the threshold for statistical significance (OR [95% CI] = 1.18 [0.45, 3.15], *p* = 0.74). A representation of this can be seen in Figure 2.

### 3.6. Screw vs. Suture Risk of Reoperation

**Figure 3:**
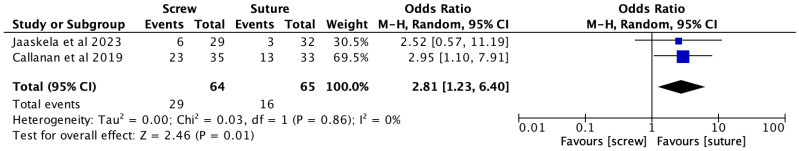
Forest Plot Comparison of Screw vs. Suture for Reoperation [14,22].

After pooling the outcomes of the studies comparing screw and suture interventions [14,22], the results revealed a significantly increased risk of screw fixation over suture fixation for reoperation (OR [95% CI] = 2.81 [1.23, 6.40], *p* = 0.01). A representation of this can be seen in Figure 3.

### 3.7. Screw vs. Suture Risk of Post-Operative Pain

**Figure 4:**
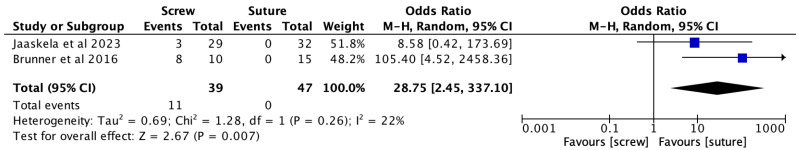
Forest Plot Comparison of Screw vs. Suture for Post-operative Pain [12,22].

After pooling the outcomes of the studies comparing screw and suture interventions [12,22], the results revealed a significantly increased risk of screw fixation over suture fixation for post-operative pain (OR [95% CI] = 28.75 [2.45, 337.10], *p* = 0.007). A representation of this can be seen in Figure 4.

### 3.8. Screw vs. Suture Risk of Instability

**Figure 5:**
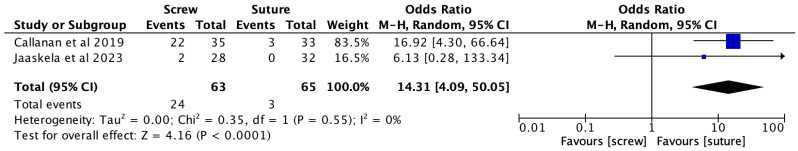
Forest Plot Comparison of Screw vs. Suture for Knee Instability [14,22].

The pooled data from the studies comparing screw and suture fixation [14,22] revealed a significantly increased risk of post-operative knee instability with screw fixation over suture fixation (OR [95% CI] = 14.31 [4.09, 50.05], *p* < 0.0001). See Figure 5.

### 3.9. ORIF vs. ARIF Risk of Arthrofibrosis

**Figure 6:**
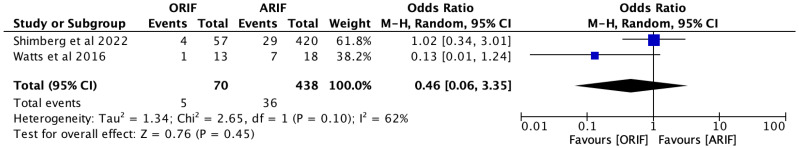
Forest Plot Comparison of ORIF vs. ARIF for Risk of Arthrofibrosis [35,41].

The pooled outcomes of the studies comparing ORIF and ARIF fixation techniques [35,41] demonstrated no difference in the risk of arthrofibrosis between ARIF and ORIF (OR [95% CI] = 0.46 [0.06, 3.35], *p* = 0.45). See Figure 6.

## 4. Discussion

We present a systematic review of the literature discussing outcomes of ORIF and ARIF techniques for the fixation of paediatric TSAFs using suture and screw materials. TSAFs are increasingly common injuries in adolescents. If left untreated, they can result in significant pain and deformity, with further complications of non-union and malunion [49]. As can be seen across all these studies, the complication rate is low, and good outcomes have been reported with all methods of fixation. There has been a general trend towards arthroscopic management, as evidenced by the current literature. This has several key advantages. First, there is reduced soft tissue dissection, which may facilitate an earlier range of motion and reduced post-operative pain. The second and perhaps most important advantage is the ability to perform a thorough inspection of the knee joint. In Shimberg et al.’s study, 7% of patients who underwent preoperative MRI had further injuries identified during fixation [50]. There are other studies that have called into question the under-sensitivity of MRI in paediatric cases. Kocher et al. found MRI had a sensitivity of 71% in partial ACL ruptures in adolescents [51]. In a larger cohort study in 2022, Dawkins et al. reported MRI scanning had moderate diagnostic ability to predict meniscal injuries with associated ACL ruptures in adolescents [52]. The performance was particularly poor with lateral meniscal tears (51% sensitivity). This is contrary to the original dogma, which states that MRI is a highly sensitive study for soft tissue near injuries. It appears true that, when ACL or meniscal injuries are present in isolation, MRI is highly sensitive and specific, but the diagnostic accuracy declines in cases where concomitant injuries are present [53]. The sensitivity declines to around 50–75% [52,54,55,56]. This could have significant implications for management. ARIF would therefore facilitate adequate inspection of the joint prior to proceeding with fixation. While concomitant injuries can be identified with an open approach, diagnostic arthroscopy would likely facilitate more thorough inspection of the joint, particularly the posteromedial and posterolateral corners. What remains unclear from the literature is whether these missed injuries would have significantly impacted the outcomes. However, diagnostic accuracy does remain a priority, and we would certainly recommend preoperative MRI in all cases, especially where the treating surgeon is planning an open approach. While it can be argued that MRI is not necessary in ARIF, we would still advise it for two reasons. First, MRI can facilitate operative planning. Second, MRI has the potential to demonstrate extension of the fracture line into the tibial plateau, which can often be missed on plain radiographs [57].

There was no clear difference in the overall complications between arthroscopic and open approaches. The traditional concern of increased risk of arthrofibrosis with ARIF appears to be unfounded. In Watts et al.’s study, prolonged time to surgery was the more significant factor in the development of arthrofibrosis [41]. This is perhaps more likely to occur in cases of ARIF, as there may be a delay until a surgeon with the appropriate skill set becomes available. Early range of motion is also important in preventing ongoing stiffness and should be encouraged post-operatively, where appropriate [58]. While ARIF provides a minimally invasive approach to fixation, along with shorter hospital stays and lower risks of infection, the surgical outcomes between ORIF and ARIF techniques remain similar. Hence, the choice of fixation technique would be heavily reliant on the experience of the surgeon.

Suture vs. screw fixation is the other key controversy in management. This review demonstrated a higher overall complication risk with screw fixation—reoperation rates were higher due to the need for metalware in screw fixation. Screw fixation can increase the risk of anterior impingement and can damage the femoral notch, but this can be mitigated with the use of a bioabsorbable screw [59]. From the studies in the review, it appears that arthroscopic suture fixation is the most common practice. Suture fixation has been shown to be biomechanically superior to screw fixation when considering the cyclical loads the knee is subjected to [60]. However, there was no difference in load required for overall failure [60]. While there is no clinical evidence to suggest one method is superior to the other with respect to fracture healing and overall outcomes, suture fixation has several additional advantages. First, sutures can be used for more comminuted MM type IV injuries; the degree of comminution may have been underestimated in preoperative imaging [60]. Second, there is a theoretical increased risk of physeal damage with screw placement, which could lead to growth arrest [50]. An all-epiphyseal approach to fixation is essential to avoid growth arrest. A review by Osti et al. also highlighted the controversy between choice of screw versus suture fixation, with screws allowing for more early mobilization and weight bearing compared to sutures [61]. However, the potential to treat small and comminuted fractures with sutures, while avoiding risks of reoperation and impaired bone growth, underlines the need to consider a risk–benefit ratio while choosing fixation materials.

This review was limited by the retrospective nature of the studies, the lack of adequate control groups in many of the studies, and the short overall follow-up. In addition, many studies had low patient numbers. This limited the depth of the meta-analysis possible. However, it is clear that TSAFs have a good prognosis if treated well, regardless of the operative approach or fixation method. We would advocate preoperative MRI in all cases, and arthroscopic suture fixation where possible, as it will allow for the most thorough inspection of the joint, and suture fixation offers superior biomechanical support and greater versatility along with a lower risk of impingement. However, we would caveat this by emphasising that all recognised approaches appear to give good outcomes with low risk of complications when performed well, and the treating surgeon should perform the procedure that best matches their skillset.

## 5. Conclusions

Overall, good outcomes are reported in TSAFs regardless of the approach or surgical fixation. There is no clear evidence to advocate one method of fixation over another. However, we would recommend arthroscopic suture fixation due to the diagnostic utility of arthroscopy and the biomechanical superiority of suture fixation. Preoperative MRI scans are essential in all cases of operative management, but surgeons should be cognisant of the limitations of MRI. Further evidence is needed to investigate the long-term outcomes and evaluate the significance of concomitant injuries that may be present.

## Figures and Tables

**Table 1:** Overview of Meyers and McKeever Classification System [4,5].

Type	Description
Type 1	Non- or minimally displaced (<3 mm)
Type 2	Minimally displaced with intact posterior hinge
Type 3a	Completely displaced involving a small portion of the eminence
Type 3b	Completely displaced involving the majority of the tibial spine
Type 4	Completely displaced, rotated, and comminuted

**Table 2:** Study characteristics table.

(Author, Year of Publication)	Title of Paper	Country of Origin	Journal of Publication	Level of Evidence
(Abdelkafy and Said, 2014) [11]	Neglected ununited tibial eminence fractures in the skeletally immature: arthroscopic management	Egypt	International Orthopaedics	4
(Brunner et al., 2016) [12]	Absorbable and non-absorbable suture fixation results in similar outcomes for tibial eminence fractures in children and adolescents	Switzerland	Knee Surgery, Sports Traumatology, Arthroscopy	3
(Caglar et al., 2021) [13]	Mid-term outcomes of arthroscopic suture fixation technique in tibial spine fractures in the paediatric population	Turkey	Ulusal Travma va Acil Cerrahi Dergisi	4
(Callanan et al., 2019) [14]	Suture Versus Screw Fixation of Tibial Spine Fractures in Children and Adolescents: A Comparative Study	USA	The Orthopaedic Journal of Sports Medicine	3
(Casalonga et al., 2010) [15]	Tibial intercondylar eminence fractures in children: The long-term perspective	France	Orthopaedics and Traumatology: Surgery and Research	4
(Chalopin et al., 2022) [16]	Arthroscopic suture-fixation of anterior tibial intercondylar eminence fractures by retensioning of the ACL and hollowing of the tibial footprint: Objective and subjective clinical results in a paediatric population	France	Orthopaedics and Traumatology: Surgery and Research	4
(Chotel et al., 2016) [17]	Cartilaginous tibial eminence fractures in children: which recommendations for management of this new entity?	France	Knee Surgery, Sports Traumatology, Arthroscopy	4
(D’ambrosio et al., 2022) [18]	Anatomical fixation of tibial intercondylar eminence fractures in children using a threaded pin with an adjustable lock	France	Orthopaedics and Traumatology: Surgery and Research	4
(Edmonds et al., 2015) [19]	Results of Displaced Paediatric Tibial Spine Fractures: A Comparison Between Open, Arthroscopic, and Closed Management	USA	Journal of Paediatric Orthopedics	3
(Furlan et al., 2010) [20]	Paediatric Tibial Eminence Fractures: Arthroscopic Treatment using K-Wire	Croatia	Scandinavian Journal of Surgery	4
(Hirschmann et al., 2009) [21]	Physeal sparing arthroscopic fixation of displaced tibial eminence fractures: a new surgical technique	Switzerland	Knee Surgery, Sports Traumatology, Arthroscopy	4
(Jaaskela et al., 2023) [22]	Long-term Outcomes of Tibial Spine Avulsion Fractures after Open Reduction with Osteosuturing Versus Arthroscopic Screw Fixation: A Multicenter Comparative Study	Italy	The Orthopaedic Journal of Sports Medicine	3
(Kieser et al., 2011) [23]	Displaced tibial intercondylar eminence fractures	New Zealand	Journal of Orthopaedic Surgery	4
(Kim et al., 2007) [24]	Arthroscopic Internal Fixation of Displaced Tibial Eminence Fracture Using Cannulated Screw	Republic of Korea	The Journal of The Korean Orthopaedic Association	4
(Kristinsson et al., 2021) [25]	Satisfactory outcomes following arthroscopic fixation of tibial intercondylar eminence fractures in children and adolescents using bioabsorbable nails	Denmark	Archives of Orthopaedic and Trauma Surgery	4
(Liljeros et al., 2009) [26]	Arthroscopic Fixation of Anterior Tibial Spine Fractures with Bioabsorbable Nails in Skeletally Immature Patients	Sweden	The American Journal of Sports Medicine	4
(Marie-Laure et al., 2008) [27]	Surgical management of type II tibial intercondylar eminence fractures in children	France	Journal of Paediatric Orthopaedics B	4
(Memisoglu et al., 2016) [28]	Arthroscopic fixation with intra-articular button for tibial intercondylar eminence fractures in skeletally immature patients	Turkey	Journal of Paediatric Orthopaedics B	4
(Momaya et al., 2017) [29]	Outcomes after arthroscopic fixation of tibial eminence fractures with bioabsorbable nails in skeletally immature patients	USA	Journal of Paediatric Orthopaedics B	4
(Najdi et al., 2016) [30]	Arthroscopic treatment of intercondylar eminence fractures with intraepiphyseal screws in children and adolescents	France	Orthopaedics and Traumatology: Surgery and Research	4
(Perugia et al., 2009) [31]	Clinical and radiological results of arthroscopically treated tibial spine fractures in childhood	Italy	International Orthopaedics (SICOT)	4
(Russu et al., 2021) [32]	Arthroscopic Repair in Tibial Spine Avulsion Fractures Using Polyethylene Terephthalate Suture: Good to Excellent Results in Paediatric Patients	Romania	Journal of Personalized Medicine	4
(Scrimshire et al., 2018) [33]	Management and outcomes of isolated paediatric tibial spine fractures	UK	Injury: International Journal of the Care of the Injured	4
(Sharma et al., 2008) [34]	An analysis of different types of surgical fixation for avulsion fractures of the anterior tibial spine	UK	Acta Orthopaedica Belgica	4
(Shimberg et al., 2022) [35]	A Multicenter Comparison of Open Versus Arthroscopic Fixation for Paediatric Tibial Spine Fractures	USA	Journal of Paediatric Orthopedics	3
(Shin et al., 2018) [36]	Clinical and radiological outcomes of arthroscopically assisted cannulated screw fixation for tibial eminence fracture in children and adolescents	Republic of Korea	BMC Musculoskeletal Disorders	4
(Sinha et al., 2017) [37]	Arthroscopic Fixation of Tibial Spine Avulsion in Skeletally Immature: The Technique	India	Journal of Orthopaedic Case Reports	4
(Tudisco et al., 2010) [38]	Intercondylar eminence avulsion fracture in children: long-term follow-up of 14 cases at the end of skeletal growth	Italy	Journal of Paediatric Orthopaedics B	4
(Uboldi et al., 2022) [39]	Arthroscopic treatment of tibial intercondylar eminence fractures in skeletally immature patients with bioabsorbable nails	Italy	La Pediatria Medica e Chirugica	4
(Vega et al., 2008) [40]	Arthroscopic Fixation of Displaced Tibial Eminence Fractures: A New Growth Plate-Sparing Method	Chile	Arthroscopy: The Journal of Arthroscopic and Related Surgery	4
(Watts et al., 2016) [41]	Open Versus Arthroscopic Reduction for Tibial Eminence Fracture Fixation in Children	USA	Journal of Paediatric Orthopedics	3
(Wiegand et al., 2014) [42]	Arthroscopic treatment of tibial spine fracture in children with a cannulated Herbert screw	Hungary	The Knee	4
(Wiktor and Tomaszewski, 2022) [43]	Results of Anterior Cruciate Ligament Avulsion Fracture by Treatment Using Bioabsorbable Nails in Children and Adolescents	Poland	Children	4
(Wouters et al., 2010) [44]	The arthroscopic treatment of displaced tibial spine fractures in children and adolescents using Mensicus Arrows^®^	The Netherlands	Knee Surgery, Sports Traumatology, Arthroscopy	4
(Xu et al., 2017) [45]	Arthroscopic fixation of paediatric tibial eminence fractures using suture anchors: A mid-term follow-up	China	Archives of Orthopaedic and Trauma Surgery	4
(Zhang et al., 2020) [46]	Arthroscopic tri-pulley Technology reduction and internal fixation of paediatric Tibial Eminence: a retrospective analysis	China	BMC Musculoskeletal Disorders	4
(Zheng et al., 2021) [47]	Arthroscopically Assisted Cannulated Screw Fixation for Treating Type III Tibial Intercondylar Eminence Fractures: A Short-Term Retrospective Controlled Study	China	Frontiers in Surgery	3
(Zhou et al., 2023) [48]	Arthroscopic percutaneous pullout suture transverse tunnel technique repair for tibial spine fractures in skeletally immature patients	China	International Orthopaedics	3

**Table 3:** MINORS Critical Appraisal Results. 0 = not reported, 1 = reported but inadequate, 2 = reported and adequate.

(Author, Year of Publication)	Item 1	Item 2	Item 3	Item 4	Item 5	Item 6	Item 7	Item 8	Item 9 ^1^	Item 10 ^1^	Item 11 ^1^	Item 12 ^1^	Total ^2^
(Abdelkafy and Said, 2014) [11]	2	1	2	2	0	2	2	0	NA ^3^	NA	NA	NA	11
(Brunner et al., 2016) [12]	2	1	0	2	0	2	2	0	2	0	2	2	15
(Caglar et al., 2021) [13]	2	1	0	1	0	1	2	0	NA	NA	NA	NA	7
(Callanan et al., 2019) [14]	2	2	1	2	0	2	2	0	2	2	2	2	19
(Casalonga et al., 2010) [15]	1	1	0	2	1	2	2	0	NA	NA	NA	NA	9
(Chalopin et al., 2022) [16]	2	1	0	2	1	2	1	0	NA	NA	NA	NA	9
(Chotel et al., 2016) [17]	2	1	0	2	0	2	2	0	NA	NA	NA	NA	9
(D’ambrosio et al., 2022) [18]	2	1	0	2	0	2	1	0	NA	NA	NA	NA	8
(Edmonds et al., 2015) [19]	2	1	1	1	0	2	2	0	2	2	1	2	16
(Furlan et al., 2010) [20]	1	2	0	2	0	2	2	0	NA	NA	NA	NA	9
(Hirschmann et al., 2009) [21]	2	0	0	2	0	2	2	0	NA	NA	NA	NA	8
(Jaaskela et al., 2023) [22]	2	2	2	2	0	2	2	0	2	2	2	2	20
(Kieser et al., 2011) [23]	1	0	0	1	0	1	2	0	NA	NA	NA	NA	5
(Kim et al., 2007) [24]	2	0	0	2	0	1	2	0	NA	NA	NA	NA	7
(Kristinsson et al., 2021) [25]	2	2	1	2	0	2	2	0	NA	NA	NA	NA	11
(Liljeros et al., 2009) [26]	2	2	1	2	0	0	1	0	NA	NA	NA	NA	8
(Marie-Laure et al., 2008) [27]	2	1	0	2	0	2	2	0	NA	NA	NA	NA	9
(Memisoglu et al., 2016) [28]	2	0	0	2	0	2	2	0	NA	NA	NA	NA	8
(Momaya et al., 2017) [29]	2	1	1	2	0	2	2	0	NA	NA	NA	NA	10
(Najdi et al., 2016) [30]	2	1	0	2	0	2	2	0	NA	NA	NA	NA	9
(Perugia et al., 2009) [31]	2	0	0	2	0	2	2	0	NA	NA	NA	NA	8
(Russu et al., 2021) [32]	2	2	2	2	1	1	2	0	NA	NA	NA	NA	12
(Scrimshire et al., 2018) [33]	2	1	0	2	0	2	1	0	NA	NA	NA	NA	8
(Sharma et al., 2008) [34]	2	1	0	2	0	2	2	0	NA	NA	NA	NA	9
(Shimberg et al., 2022) [35]	2	2	1	2	0	1	2	0	2	2	2	2	18
(Shin et al., 2018) [36]	2	2	0	2	0	2	2	0	NA	NA	NA	NA	10
(Sinha et al., 2017) [37]	1	0	0	2	0	1	2	0	NA	NA	NA	NA	6
(Tudisco et al., 2010) [38]	2	1	0	2	0	2	2	0	1	2	0	0	12
(Uboldi et al., 2022) [39]	2	1	0	2	0	2	2	0	NA	NA	NA	NA	9
(Vega et al., 2008) [40]	2	1	0	2	0	1	2	0	NA	NA	NA	NA	8
(Watts et al., 2016) [41]	2	2	1	2	0	1	2	0	2	2	2	2	18
(Wiegand et al., 2014) [42]	2	1	1	2	0	1	2	0	NA	NA	NA	NA	9
(Wiktor and Tomaszewski, 2022) [43]	2	1	0	2	0	2	2	0	NA	NA	NA	NA	9
(Wouters et al., 2010) [44]	2	2	1	2	0	2	2	0	NA	NA	NA	NA	11
(Xu et al., 2017) [45]	2	2	1	2	1	2	2	0	NA	NA	NA	NA	12
(Zhang et al., 2020) [46]	2	2	1	2	1	2	2	0	NA	NA	NA	NA	12
(Zheng et al., 2021) [47]	1	1	0	2	0	2	2	0	NA	NA	NA	NA	8
(Zhou et al., 2023) [48]	2	2	1	2	0	2	2	0	NA	NA	NA	NA	11

^1^ Items 9–12 were only considered for comparative studies. Studies with no comparison group were critically appraised using items 1–8 only. ^2^ The maximum MINORS score was 16 for non-comparative studies and 24 for comparative studies. ^3^ NA = Not Applicable.

**Table 4:** Participant Characteristics. NR = Not Reported.

(Author, Year of Publication)	Number of Participants	Mean Age	Meyers and McKeever Classification	Surgical Approach	Fixation Method	Mean Follow-Up Time
(Abdelkafy and Said, 2014) [11]	13	10 ± 2.6	I: 0 II: 0 III: 13 IV: 0	Arthroscopic: 13 Open: 0	Screw: 0 Suture: 13	10.8 ± 6.8 months
(Brunner et al., 2016) [12]	25	Group A: 11.1 ± 3.3 Group B: 11.7 ± 3.3	I: 0 II: 11 III: 14 IV: 0	Arthroscopic: 25 Open: 0	Screw: 10 (non-absorbable suture with screw; Group B) Suture: 15 (absorbable with transosseus fixation; Group A)	Group A: 28.1 ± 4.6 months Group B: 47.4 ± 20.7 months
(Caglar et al., 2021) [13]	28	14.2 (8–18)	I: 0 II: 16 III: 10 IV: 2	Arthroscopic: 28 Open: 0	Screw: 0 Suture: 28	4.64 years
(Callanan et al., 2019) [14]	68	11.8 ± 2.99	I: 0 II: 14 III: 50 IV: 0	Arthroscopic: 68 Open: 0	Screw: 35 Suture: 33	26 (17–47) months
(Casalonga et al., 2010) [15]	32	12.0	I: 8 II: 17 III: 5 IV: 2	Arthroscopic: 0 Open: 7 Conservative: 25	Screw: 3 Suture: 4	14 years and 11 months (5–21 years)
(Chalopin et al., 2022) [16]	17	12 (7–15)	I: 0 II: 5 III: 9 IV: 3	Arthroscopic: 17 Open: 0	Screw: 0 Suture: 17 (Single sutures: 11, Double sutures: 6)	28 months (16–48 months)
(Chotel et al., 2016) [17]	15	6.5 ± 1.4	I: 0 II: 3 III: 6 IV: 6	Arthroscopic: 6 Open: 0 Mixed: 4 Conservative: 2	Screw: 0 Suture: 8	4.6 years (1–18.5)
(D’ambrosio et al., 2022) [18]	34	11.5 ± 2.7	I: 0 II: 19 III: 12 IV: 3	Arthroscopic: 34 Open: 0	Screw: 34 Suture: 0	8.8 ± 6 years
(Edmonds et al., 2015) [19]	18	Arthroscopic: 18.3 ± 2.0 Open: 18.2 ± 3.0 Conservative: 17.4 ± 5.0	NR	Arthroscopic: 5 Open: 7 Conservative: 6	Screw: 0 Suture: 12	Arthroscopic: 5.6 ± 2.0 years Open: 6.8 ± 2.0 years Conservative: 5.8 ± 2.0 years
(Furlan et al., 2010) [20]	10	15 (12–17)	I: 0 II: 5 III: 4 IV: 1	Arthroscopic: 10 Open: 0	NR (K-wire fixation)	42 (9–78) months
(Hirschmann et al., 2009) [21]	6	14 ± 2	I: 0 II: 2 III: 3 IV: 1	Arthroscopic: 6 Open: 0	Screw: 6 Suture: 0	5 ± 2 years
(Jaaskela et al., 2023) [22]	61	11.2 ± 2.6	I: 1 II: 26 III: 34 IV: 0	Arthroscopic: 29 Open: 32	Screw: 29 Suture: 32	87.0 ± 47.1 months
(Kieser et al., 2011) [23]	9	12 (6–15)	I: 0 II: 2 III: 7 IV: 0	Arthroscopic: 2 Open: 7	Screw: 2 Suture: 6	45 (6–260) weeks
(Kim et al., 2007) [24]	10	10.5 (7–13)	I: 0 II: 4 III: 6 IV: 0	Arthroscopic: 10 Open: 0	Screw: 10 Suture: 0	22.4 (12–81) months
(Kristinsson et al., 2021) [25]	13	11 (4–15)	I: 0 II: 9 III: 2 IV: 2	Arthroscopic: 13 Open: 0	Screw: 13 Suture: 0	6.5 (1–10) years
(Liljeros et al., 2009) [26]	13	11 (7–15)	I: 0 II: 1 III: 12 IV: 0	Arthroscopic: 13 Open: 0	Screw: 13 Suture: 0	NR
(Marie-Laure et al., 2008) [27]	17	12.1 (6–16)	I: 0 II: 17 III: 0 IV: 0	Arthroscopic: 0 Open: 17	NR	3 (0.5–7) years
(Memisoglu et al., 2016) [28]	11	12.2 (10–16)	I: 0 II: 1 III: 9 (A), 1 (B) IV: 1	Arthroscopic: 11 Open: 0	Screw: 0 Suture: 0 Both: 11 (+ Endobutton)	69 (60–84) months
(Momaya et al., 2017) [29]	7	11.6 (8–15)	I: 0 II: 1 III: 6 IV: 0	Arthroscopic: 7 Open: 0	Screw: 7 Suture: 0	31 (24–36) months
(Najdi et al., 2016) [30]	24	11 (6–15)	I: 0 II: 15 III: 9 IV: 0	Arthroscopic: 24 Open: 0	Screw: 24 Suture: 0	2 (1.5–3) years
(Perugia et al., 2009) [31]	10	13.5 (12–15)	I: 0 II: 3 III: 7 IV: 0	Arthroscopic: 10 Open: 0	Screw: 0 Suture: 10	85.8 (20–188) months
(Russu et al., 2021) [32]	12	14.3 ± 2.1	I: 0 II: 0 III: 12 IV: 0	Arthroscopic: 12 Open: 0	Screw: 0 Suture: 12	6 months
(Scrimshire et al., 2018) [33]	40	11.8	I: 3 II: 13 III: 24 IV: 0	Arthroscopic: 0 Open: 30 Conservative: 10	Screw: 30 Suture: 0	36 months
(Sharma et al., 2008) [34]	14 (children), 11 (adults)	13 (8–16)	I: 0 II: 0 III: 19 IV: 6	Arthroscopic: 0 Open: 24	Screw: 7 (children), 6 (adults) Suture: 6 (children), 3 (adults) Stainless steel loop: 2 (children), 2 (adults)	44 months
(Shimberg et al., 2022) [35]	477	Arthroscopic: 12.1 Open: 12.5	I: 14 II: 211 III: 252 IV: 0	Arthroscopic: 420 Open: 57	NR	1.12 years
(Shin et al., 2018) [36]	27	10.1 ± 2.2	I: 0 II: 12 III: 13 IV: 2	Arthroscopic: 27 Open: 0	Screw: 27 Suture: 0	3.9 ± 2.2 years
(Sinha et al., 2017) [37]	10	12.1 ± 1.9	NR	Arthroscopic: 10 Open: 0	Screw: 0 Suture: 10	12 months
(Tudisco et al., 2010) [38]	14	12.25 (7–16)	I: 4 II: 3 III: 7 IV: 0	Arthroscopic: 6 Open: 1 Conservative: 7	Screw: 0 Suture: 14	29 (12–42) years
(Uboldi et al., 2022) [39]	19	10 (6–13)	I: 0 II: 5 III: 14 IV: 0	Arthroscopic: 19 Open: 0	Screw: 19 Suture: 0	27 (6–60) months
(Vega et al., 2008) [40]	7	11.8	I: 0 II: 0 III: 5 IV: 2	Arthroscopic: 7 Open: 0	Screw: 0 Suture: 0 Both: 7	6 (6–24) months
(Watts et al., 2016) [41]	31	Arthroscopic group: 12.9 (7–18) Open group: 11.5 (7–16)	NR	Arthroscopic: 18 Open: 13	Screw: 17 Suture: 11 Both: 3	Arthroscopic: 13.9 (3–33) months Open: 12.7 (3–50) months
(Wiegand et al., 2014) [42]	8 (+4 treated conservatively)	12.5	I: 4 II: 3 III: 5 IV: 0	Arthroscopic: 8 Open: 0 Conservative: 4	Screw: 8 Suture: 0	1 year
(Wiktor and Tomaszewski, 2022) [43]	17	10.1 (5–15.2)	I: 0 II: 5 III: 10 IV: 2	Arthroscopic: 10 Open: 7	Screw: 17 Suture: 0	28 ± 21.9 months
(Wouters et al., 2010) [44]	12	12.0 (6–15)	NR	Arthroscopic: 12 Open: 0	NR	3–10 years
(Xu et al., 2017) [45]	20	15.3 (13–17)	I: 0 II: 10 III: 6 IV: 4	Arthroscopic: 20 Open: 0	Screw: 0 Suture: 20	43.4 (40–47) months
(Zhang et al., 2020) [46]	21	12.5 (8–16)	I: 0 II: 14 III: 3 (A), 4 (B) IV: 0	Arthroscopic: 21 Open: 0	Screw: 0 Suture: 21	28.4 ± 5.6 months
(Zheng et al., 2021) [47]	Group 1 (arthroscopically assisted cannulated screw fixation) = 12 Group 2 (open reduction and cannulated screw internal fixation) = 10	Group 1: 10.94 ± 2.00 Group 2: 10.85 ± 1.53	I: 0 II: 12 III: 22 IV: 0	Arthroscopic: 12 Open: 10	Screw: 22 Suture: 0	27.5 (12–58) months
(Zhou et al., 2023) [48]	Group 1 (transtibial pullout suture technique) = 21 Group 2 (percutaneous pullout suture transverse tunnel) = 20	Group 1: 12.5 ± 2.6 Group 2: 11.3 ± 2.9	I: 0 II: 19 III: 22 IV: 0	Arthroscopic: 41 Open: 0	Screw: 0 Suture: 41	Group 1: 33.27 ± 4.18 months Group 2: 34.15 ± 3.65 months

**Table 5:** Study Outcomes. International Knee Documentation Committee (IKDC), Visual Analog Scale (VAS), Association pour la Recherche et la Promotion de l’Étude du Genou (ARPEGE), Knee Injury and Osteoarthritis Outcome Score (KOOS), Activities of Daily Living (ADL), Quality of Life (QOL). NR = Not Reported.

(Author, Year of Publication)	Pre-Surgery IKDC Score	Post-Surgery IKDC Score	Pre-Surgery Lysholm Score	Post-Surgery Lysholm Score	Other Outcomes	
					Pre-Surgery	Post-Surgery
(Abdelkafy and Said, 2014) [11]	Objective: Grade B (1), Grade C (10), Grade D (2) Subjective: 15.4 ± 4.2	Objective: Grade A (12), Grade B (1) Subjective: 80.5 ± 16.7	3.8 ± 2.5	91.2 ± 8.9	VAS: 8.5 ± 1.2 (pain)	VAS: 9.6 ± 0.5 (operation satisfaction), 0.4 ± 0.5 (pain)
(Brunner et al., 2016) [12]	NR	Objective Group A: Grade A (10), Grade B (5) Objective Group B: Grade A (7), Grade B (3) Subjective: NA	NR	Group A: 94.1 ± 8.1 Group B: 90.1 ± 10.2	NR	Rollimeter difference to ipsilateral knee (mm): Group A: 0.5 ± 0.8 Group B: 0.5 ± 0.7
(Caglar et al., 2021) [13]	NR	Objective: NR Subjective: 6 months: 82.3 (68–91); 12 months: 91.4 (81–100); 24 months: 95.7 (89–100)	NR	NR	NR	NR
(Callanan et al., 2019) [14]	NR	NR	NR	NR	NR	Time to radiographic union: 2.1 years (suture); 4.3 years (screw)
(Casalonga et al., 2010) [15]	NR	Objective: Grade A (4), Grade B (4), Grade C (4), Grade D (1) Subjective: 91 (mailed, *n* = 10), 81 (at follow-up, *n* = 13)	NR	NR	NR	ARPEGE Score: 8.3
(Chalopin et al., 2022) [16]	NR	Objective: Grade A (14), Grade B (3) Subjective: 97 ± 2.46	NR	99.1 ± 1.62	NR	NR
(Chotel et al., 2016) [17]	NR	Objective: Grade A (9), Grade B (3), Grade C (1) Subjective: 97 (91–100)	NR	97.36 (94–100)	NR	NR
(D’ambrosio et al., 2022) [18]	NR	Objective: NR Subjective: 93.8 ± 6.4	NR	93.1 ± 9.8	NR	Average return to sport time: 9.1 ± 9.5 months Average Tegner Score: 5.6 ± 1.5
(Edmonds et al., 2015) [19]	NR	NR	NR	Arthroscopic: 95 Open: 97.4 Conservative: 86	NR	Pain (0–10): Arthroscopic: 0.2 Open: 0.7 Conservative: 2.7
(Furlan et al., 2010) [20]	NR	Objective: Grade A (8), Grade B (2) Subjective: 96 (85–100)	NR	NR	NR	NR
(Hirschmann et al., 2009) [21]	NR	Objective: Grade A (5), Grade B (1) Subjective: 197 ± 4	NR	97 ± 3	Tegner Score: 8 (6–9)	VAS: 0.5 ± 0.8 (pain), 9.5 ± 1.5 (satisfaction) Tegner Score: 8 (6–9)
(Jaaskela et al., 2023) [22]	NR	Objective: NR Subjective: 93.1 ± 13.5 (open osteosuture), 90.4 ± 14.5 (arthroscopic screw)	NR	NR	NR	Time to return to sport (weeks): 8.0 (8–12) (open osteosuture), 21.0 (12–36.3) (arthroscopic screw)
(Kieser et al., 2011) [23]	NR	NR	NR	NR	NR	NR
(Kim et al., 2007) [24]	NR	NR	NR	96.3 (92.6–99.0)	NR	NR
(Kristinsson et al., 2021) [25]	NR	NR	NR	NR	NR	KOOS Scores: (1) Pain: 100 (19–100) (2) Symptoms: 91.0 (54–100) (3) ADL: 100 (22–100) (4) Sport: 90.0 (0–100) (5) QOL: 88.0 (13–100) EQ5D-5L index value: 1.0 (0.225–1) EQ5D-5L VAS–92.0 (50–100)
(Liljeros et al., 2009) [26]	NR	NR	NR	93.69	Activity Level (1–3): 2 (1–3)	Activity Level (1–3): 2 (1–3)
(Marie-Laure et al., 2008) [27]	NR	NR	NR	99.7 (95–100)	NR	NR
(Memisoglu et al., 2016) [28]	NR	Objective: Grade A (7), Grade B (4) Subjective: 94.3 (85–100)	NR	95.7 ± 6.6	NR	NR
(Momaya et al., 2017) [29]	NR	Objective: NR Subjective: 97.3 ± 3.5	NR	95.6 ± 5.2	NR	NR
(Najdi et al., 2016) [30]	NR	NR	NR	99.1 ± 1.9	NR	NR
(Perugia et al., 2009) [31]	NR	Objective: Grade A (3), Grade B (4), Grade C (3) Subjective: 92.4 ± 3.3	NR	95.9 ± 2.9	NR	NR
(Russu et al., 2021) [32]	Objective: NR Subjective: 33.4 ± 23.3	Objective: NR Subjective: 84.2 ± 14.3	53.7 ± 17.3	87.7 ± 9.9	Tegner Score: 3.8 ± 1.1	Tegner Score: 6.7 ± 2.2
(Scrimshire et al., 2018) [33]	NR	NR	NR	Operative: 94 (washer used = 92, no washer used = 96) Non-operative: 95	NR	Cincinnati Score: Operative: 96 Non-operative: 96
(Sharma et al., 2008) [34]	NR	NR	NR	Screw and wire (non-absorbable): 89 (69–100) Suture (absorbable): 100 (85–100)	NR	NR
(Shimberg et al., 2022) [35]	NR	NR	NR	NR	NR	NR
(Shin et al., 2018) [36]	NR	NR	NR	94.8 ± 6.8	NR	NR
(Sinha et al., 2017) [37]	NR	NR	50.8 ± 1.4	96.3 ± 2.9	NR	NR
(Tudisco et al., 2010) [38]	NR	Objective: Grade A (2), Grade B (11), Grade C (1) Subjective: NR	NR	NR	NR	NR
(Uboldi et al., 2022) [39]	NR	Objective: Grade A (18), Grade B (19) Subjective: 88.45 (80–95)	NR	NR	Tegner Activity Scale: 5.51 (3–7)	Tegner Activity Scale: 5.61 (4–7)
(Vega et al., 2008) [40]	NR	Objective: Grade A (4), Grade B (3) Subjective: 92 (86–98)	29	94	NR	NR
(Watts et al., 2016) [41]	NR	NR	NR	NR	NR	NR
(Wiegand et al., 2014) [42]	NR	NR	NR	Conservative (Type I): 97.00 Arthroscopic (Type II): 94.97 Arthroscopic (Type III): 94.20	NR	NR
(Wiktor and Tomaszewski, 2022) [43]	NR	Objective: NR Subjective: 84.64 ± 3.10	NR	96.64 ± 4.54	NR	NR
(Wouters et al., 2010) [44]	NR	NR	NR	NR	NR	NR
(Xu et al., 2017) [45]	Objective: Grade C (15), Grade D (5) Subjective: NR	Objective: Grade A (13), Grade B (7) Subjective: NR	57.5 ± 11.2	91.0 ± 7.2	Tegner Score: 4.6 ± 1.4	Tegner Score: 8.0 ± 1.7
(Zhang et al., 2020) [46]	Objective: NR Subjective: 43.1 ± 13.2	Objective: NR Subjective: 83.8 ± 6.3	48.3 ± 6.21	87.1 ± 9.8	NR	NR
(Zheng et al., 2021) [47]	NR	Objective: NR Subjective: Group 1: 92.06 ± 3.55 Group 2: 86.07 ± 5.81	NR	Group 1: 93.33 ± 3.55 Group 2: 86.20 ± 4.52	NR	Tegner Score: Group 1: 7.75 ± 0.87 Group 2: 6.40 ± 0.52
(Zhou et al., 2023) [48]	Objective: NR Subjective: Group 1: 46.16 ± 12.57 Group 2: 47.27 ± 11.87	Objective: NR Subjective: Group 1: 90.15 ± 8.12 Group 2: 92.14 ± 7.89	Group 1: 43.23 ± 9.54 Group 2: 41.62 ± 10.15	Group 1: 91.08 ± 7.65 Group 2: 92.54 ± 9.17	Tegner Score: Group 1: 3.26 ± 1.54 Group 2: 3.02 ± 1.34 VAS Score: Group 1: 4.86 ± 0.53 Group 2: 5.13 ± 0.71	Tegner Score: Group 1: 5.76 ± 1.12 Group 2: 5.52 ± 1.01 VAS Score: Group 1: 1.23 ± 0.41 Group 2: 1.31 ± 0.51

**Table 6:** Complications. NR = Not Reported.

(Author, Year of Publication)	Wound Infection	Post-Surgical Pain	Stiffness	Instability	Arthrofibrosis	Reoperation	Leg Length Discrepancy	Deep Venous Thrombosis
(Abdelkafy and Said, 2014) [11]	1 (superficial)	0	0	0	0	0	0	0
(Brunner et al., 2016) [12]	0	Group B: 8 (pain around screw)	0	0	Group A: 3 Group B: 1	0	0	0
(Caglar et al., 2021) [13]	0	0	1	1	1	1	0	0
(Callanan et al., 2019) [14]	0	0	Suture: 8 Screw: 11	Suture: 3 Screw: 22	Suture: 8 Screw: 11	Suture: 13 Screw: 23	0	0
(Casalonga et al., 2010) [15]	0	0	3 (Type II)	0	0	1	0	1
(Chalopin et al., 2022) [16]	0	0	0	0	0	0	0	0
(Chotel et al., 2016) [17]	0	0	1	0	0	4	6	0
(D’ambrosio et al., 2022) [18]	0	0	0	5	0	0	0	0
(Edmonds et al., 2015) [19]	0	Conservative: 3	0	0	0	0	0	0
(Furlan et al., 2010) [20]	0	0	0	0	0	0	0	0
(Hirschmann et al., 2009) [21]	0	0	0	0	0	0	0	0
(Jaaskela et al., 2023) [22]	0	Arthroscopic screw: 3	0	Arthroscopic screw: 2	0	Arthroscopic screw: 6 Open osteosuture: 3	0	0
(Kieser et al., 2011) [23]	0	0	0	0	1	2	0	0
(Kim et al., 2007) [24]	0	0	0	0	0	0	0	0
(Kristinsson et al., 2021) [25]	0	0	0	0	0	0	0	0
(Liljeros et al., 2009) [26]	0	1	0	1	0	0	0	0
(Marie-Laure et al., 2008) [27]	0	0	0	0	0	0	0	0
(Memisoglu et al., 2016) [28]	0	0	0	0	0	2	0	0
(Momaya et al., 2017) [29]	0	0	0	0	1	0	0	0
(Najdi et al., 2016) [30]	0	0	1	0	0	0	0	0
(Perugia et al., 2009) [31]	0	0	0	0	0	0	0	0
(Russu et al., 2021) [32]	NR	NR	NR	NR	NR	NR	NR	NR
(Scrimshire et al., 2018) [33]	0	1	5	0	0	9	0	0
(Sharma et al., 2008) [34]	1	0	0	6	0	1	0	0
(Shimberg et al., 2022) [35]	Arthroscopic: 2 (0.5%)	0	0	0	Arthroscopic: 29 (6.9%) Open: 4 (7.0%)	Arthroscopic: 90 (21%) Open: 18 (32%)	Arthroscopic: 6 (1.4%)	0
(Shin et al., 2018) [36]	0	0	1	0	0	0	10	0
(Sinha et al., 2017) [37]	NR	NR	NR	NR	NR	NR	NR	NR
(Tudisco et al., 2010) [38]	0	0	0	Conservative: 1	2	Conservative: 1	0	0
(Uboldi et al., 2022) [39]	0	0	0	0	0	0	0	0
(Vega et al., 2008) [40]	NR	NR	NR	NR	NR	NR	NR	NR
(Watts et al., 2016) [41]	0	0	0	0	Arthroscopic: 7 Open: 1	10	0	0
(Wiegand et al., 2014) [42]	0	0	0	0	1	0	0	0
(Wiktor and Tomaszewski, 2022) [43]	0	0	4	0	0	0	0	0
(Wouters et al., 2010) [44]	0	0	0	0	0	1	0	0
(Xu et al., 2017) [45]	0	0	0	0	0	0	0	0
(Zhang et al., 2020) [46]	0	0	0	0	0	0	0	0
(Zheng et al., 2021) [47]	0	0	0	0	0	0	0	0
(Zhou et al., 2023) [48]	0	0	0	0	0	0	0	0

## Appendix A

**Table A1:** Detailed Search Strategy with search terms. ? = wild card used to account for missing characters, * = truncation tool used to account for alternative forms of the root word.

Tibial Spine Avulsion Fracture AND	Surgical Management AND	Paediatric Patients
(Tibial OR Tibia) AND (spine OR eminence OR inter?condylar OR inter?condyle) AND fracture OR avulsion Tibial eminence avulsion Tibial eminence fracture Intercondylar fracture Intercondylar avulsion Anterior cruciate ligament avulsion ACL avulsion	Surgery Surgical treatment Surgical management Operative treatment Operative management Surgical technique Management Treatment Fracture fixation Surgical fixation	Paediatric * Child * Youth High school Adolescent * Paediatric surgery Juvenile
